# A Nanobody‐on‐Quantum Dot Displacement Assay for Rapid and Sensitive Quantification of the Epidermal Growth Factor Receptor (EGFR)

**DOI:** 10.1002/anie.202207797

**Published:** 2022-07-08

**Authors:** Ruifang Su, Yu‐Tang Wu, Sofia Doulkeridou, Xue Qiu, Thomas Just Sørensen, Kimihiro Susumu, Igor L. Medintz, Paul M. P. van Bergen en Henegouwen, Niko Hildebrandt

**Affiliations:** ^1^ nanoFRET.com Laboratoire COBRA (UMR6014 & FR3038) Université de Rouen Normandie, CNRS, INSA Normandie Université 76000 Rouen France; ^2^ Nano-Science Center & Department of Chemistry University of Copenhagen Universitetsparken 5 2100 Copenhagen Denmark; ^3^ Université Paris-Saclay, CEA, CNRS Institute for Integrative Biology of the Cell (I2BC) 91198 Gif-sur-Yvette France; ^4^ Cell Biology Neurobiology and Biophysics Department of Biology Science Faculty Utrecht University 3508 TB Utrecht The Netherlands; ^5^ Princess Maxima Center Heidelberglaan 25 3584CS Utrecht The Netherlands; ^6^ Key Laboratory of Marine Drug Ministry of Education School of Medicine and Pharmacy Ocean University of China 266003 Qingdao China; ^7^ Laboratory for Marine Drugs and Bioproducts of Qingdao National Laboratory for Marine Science and Technology 266237 Qingdao China; ^8^ Jacobs Corporation Hanover MD 21076 USA; ^9^ Optical Sciences Division, Code 5600, Code 6900 U.S. Naval Research Laboratory Washington DC 20375 USA; ^10^ Center for Bio/Molecular Science and Engineering, Code 6900 U.S. Naval Research Laboratory Washington DC 20375 USA; ^11^ Department of Chemistry Seoul National University Seoul 08826 South Korea

**Keywords:** Diagnostics, EGFRvIII, FRET, Lanthanides, Nanoparticles

## Abstract

Biosensing approaches that combine small, engineered antibodies (nanobodies) with nanoparticles are often complicated. Here, we show that nanobodies with different C‐terminal tags can be efficiently attached to a range of the most widely used biocompatible semiconductor quantum dots (QDs). Direct implementation into simplified assay formats was demonstrated by designing a rapid and wash‐free mix‐and‐measure immunoassay for the epidermal growth factor receptor (EGFR). Terbium complex (Tb)‐labeled hexahistidine‐tagged nanobodies were specifically displaced from QD surfaces via EGFR‐nanobody binding, leading to an EGFR concentration‐dependent decrease of the Tb‐to‐QD Förster resonance energy transfer (FRET) signal. The detection limit of 80±20 pM (16±4 ng mL^−1^) was 3‐fold lower than the clinical cut‐off concentration for soluble EGFR and up to 10‐fold lower compared to conventional sandwich FRET assays that required a pair of different nanobodies.

## Graphical abstract

Simple is powerful: Terbium complexes labeled with hexahistidine‐tagged nanobodies were displaced from quantum dots via EGFR‐nanobody binding. This simple concept resulted in a rapid and wash‐free single‐nanobody FRET immunoassay with picomolar limits of detection.

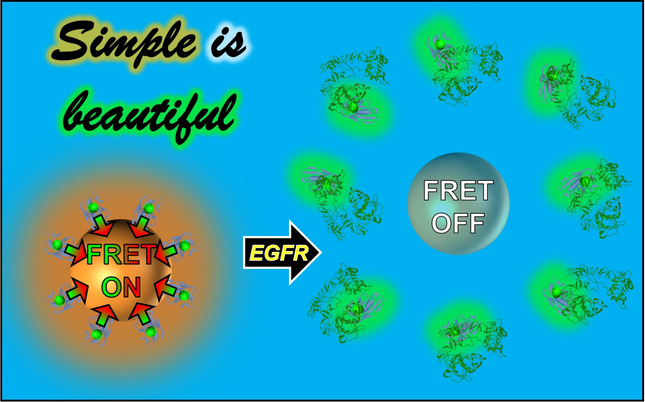

Ultrasensitive bioassays are essential for the quantification of different biomarkers in clinical diagnostics. Assays based on Förster resonance energy transfer (FRET) are of particular interest, because they are highly sensitive and very easy to perform as they do not require any separation or washing steps.[^1^, ^2^] However, FRET is only functional for donor‐acceptor distances below circa 10 nm,^[3]^ which makes it a challenging endeavor for the development of immunochemical biosensors based on nanoparticles (NPs).^[4]^

Nanobodies (NBs, or variable domains of the heavy chain of heavy‐chain‐only antibodies—VHH) are genetically engineered small (≈15 kDa with a cylindrical shape of ≈2.5 nm diameter and ≈4 nm height) antibodies that have found frequent application in molecular imaging, clinical diagnostics, and disease therapy.[^5^, ^6^, ^7^, ^8^, ^9^, ^10^, ^11^] Their much smaller size compared to intact IgG antibodies (≈150 kDa) combined with simple expression, high stability, high solubility, and many chemical functionalization strategies provide NBs with several advantages for biosensing and bioimaging.[^6^, ^9^, ^10^] To design functional nanobiosensors, NBs have been bioconjugated to various nanomaterials,[^6^, ^12^] including gold NPs,^[13]^ graphene oxide,^[14]^ lanthanide NPs,^[15]^ gold‐platinum core–shell NPs,^[16]^ quantum dots (QDs),[^17^, ^18^] and porphyrin‐based metal organic framework NPs.^[19]^ QDs are arguably the most applied nanomaterials for FRET biosensing,[^20^, ^21^, ^22^, ^23^] and NBs have been used to develop QD‐to‐dye,^[24]^ QD‐to‐QD,^[25]^ fluorescent protein‐to‐QD,^[26]^ and lanthanide‐to‐QD[^27^, ^28^, ^29^] FRET immunosensors.

Specific attachment and orientation of small antibodies on QDs for improved immunotargeting has been demonstrated with cysteine (Cys) tagged NBs,^[29]^ hexahistidine (His_6_) and Cys tagged albumin‐binding domain‐derived affinity proteins (ADAPTs),^[30]^ His_6_ tagged artificial repeat proteins (αReps),^[31]^ and split protein (SpyCatcher/SpyTag) tagged short‐chain variable fragments (scFv).^[32]^ Beyond these proofs‐of‐concept, the translation of NB‐QD conjugates into standard probes that can be adapted to a broad range of biosensing approaches requires experimental comparison of different straightforward NB‐QD bioconjugation methods using the same type of NB and commercially available QDs. Such one‐to‐one evaluation of bioassay performance can provide important knowledge of how NB‐QD bioconjugates can be efficiently implemented into diverse applications. Another important challenge to demonstrate the benefits of NB‐QD based immunosensors for daily use in biological, biochemical, or chemical laboratories is to make use of that knowledge and exploit NB‐tags and QDs for the development of novel, simple, and efficient biosensing formats.

With these two major goals in mind, we implemented three widely used bioconjugation tags, namely His_6_, Cys, and biotin, into the C‐termini of two different NBs (EgB4 and EgA1) against the epidermal growth factor receptor (EGFR) as representative protein biomarker. The functionalized NBs were then used to prepare NB‐QD bioconjugates with commercially available and widely used QDs of two different colors (QDot 625 and QDot 705, Thermo Fisher) and with three common surface coatings, namely compact zwitterionic ligands (CL4),^[33]^ amino‐polyethylene‐glycol (PEG), and streptavidin (sAv). To demonstrate the biosensing functionality of this versatile bioconjugation toolkit, we compared the various NB‐QD bioconjugates in wash‐free and rapid FRET sandwich immunoassays for the quantification of EGFR. We then developed a new biosensing concept, in which His_6_‐tagged NBs were displaced from the QD surface by non‐competitive binding of NB to EGFR. This new assay format, which required only a single type of NB and no QD bioconjugation, was applied for the quantification of soluble EGFR (sEGFR, a prognostic and predictive biomarker for metastatic breast cancer)^[34]^ and soluble EGFR variant III (sEGFRvIII, a prognostic biomarker for glioblastoma).^[35]^ The NB‐displacement assay significantly decreases cost and labor (for antibody screening and production and bioconjugation), strongly facilitates assay‐kit assembly and storage (only one type of Tb‐NB conjugate and one type of unlabeled QD), provides rapid (mix‐and‐measure) analysis, and can quantify relevant biomarkers at clinically relevant concentrations.

For an easier understanding of the different materials and material combinations, we introduced specific abbreviations (Table 1). The two different NBs do not compete for binding to EGFR (Supporting Figure S1) and can thus be used to detect the soluble EGFR ectodomain in immunological sandwich assays. NB1 binds sEGFR and sEGFRvIII in the cleft formed between domains **II** and **III**,[^36^, ^37^] whereas NB2 only binds to domains **I** and **II** of sEGFR.[^36^, ^38^] Conjugation to the different tags did not affect their affinity for EGFR (Supporting Figure S2 and Table S1). The spectral properties of the Lumi4‐Tb (Tb) FRET donor and QD acceptors used for the FRET immunoassays are shown in Supporting Figure S3. Tb FRET donor conjugates were prepared using amino‐reactive chemistry between Lumi4‐Tb‐n‐hydroxysuccinimide(NHS) and NB1 (Tb‐NB1) or NB1‐H (Tb‐NB1‐H).^[29]^ QD FRET acceptor conjugates were prepared depending on the different tags on NB2: *i) NB2‐H‐QD625‐CL4*: QD625‐CL4 were mixed in a 1 : 20 molar ratio with NB2‐H, which resulted in efficient metal‐affinity mediated self‐assembly to the Zn‐rich QD surface after ≈30 min.^[39]^ Considering the 2.5 nm diameter of NBs,^[9]^ 20 NB2 should take approximately 50 nm^2^ of space on the ≈265 nm^2^ surface of the QD (*A*=4π*r* ^2^ with *r*=4.6 nm), which can be considered as low enough to avoid steric hindrance and allow for efficient self‐assembly. *ii) NB2‐B‐QD705‐sAv*: NB2‐B was attached to QD705‐sAv via the strong biotin‐sAv interaction,^[40]^ also by simple mixing for ≈30 min in a 20 : 1 molar ratio. Considering the four biotin binding sites of sAv and the number of circa 6 to 10 sAv per QD, there should be sufficient binding sites for the 20 NB2‐B per QD705‐sAv. *iii) NB2‐C‐QD705‐PEG*: Bioconjugation of QD705‐PEG was more complex and was performed via N‐ϵ‐maleimidocaproyl‐oxysulfosuccinimide ester (sulfo‐EMCS) crosslinkers that were first attached to the QDs and then reacted with a ≈90‐fold molar excess of NB2‐C for 6 h followed by separation of unbound compounds.^[30]^

**Table 1:** Materials (and their short names) used for FRET assay development.^[a]^

**FRET donor**	**Short name**
Lumi4‐Tb	Tb

**FRET acceptors**	**Short name**
QDot625‐CL4	QD625‐CL4
QDot705 streptavidin	QD705‐sAv
QDot705 amino‐PEG	QD705‐PEG

**Nanobodies**	**Short name**
EgA1 (no tag)	NB1
EgA1‐His_6_	NB1‐H
EgB4‐Cysteine	NB2‐C
EgB4‐Biotin	NB2‐B
EgB4‐His_6_	NB2‐H

[a] More detailed information can be found in the Supporting Information.

Bioconjugation strategies (i) and (ii) have significant advantages. Preparation is rapid and simple and does not require separation because both metal‐affinity mediated self‐assembly and biotin‐sAv binding are very efficient (quantitative labeling). Thus, the molar ratio of NB and QD can be considered as the labeling ratio (20 NB per QD). Moreover, NB‐QD conjugates can be prepared directly before the immunoassays, which avoids storage problems and guarantees reproducible NB‐QD bioconjugates for each assay. The longer and more complex bioconjugation procedure (iii) prevents preparation directly before the biosensing experiments. The actual labeling ratio is also more difficult to estimate because absorption measurements at 280 nm (more than 200‐fold higher absorption of QDs compared to NBs) are inaccurate. However, more important than the actual number of NBs is the accessibility of the NBs on the QDs, which we directly evaluated by binding‐saturation in the FRET assays (vide infra).

The analytical performance of the different NB‐QD bioconjugates was compared in time‐gated (TG) FRET sandwich assays for sEGFR quantification (Figure 1A) using the clinical diagnostic TG‐FRET plate reader *KRYPTOR compact PLUS* (Thermo Fisher Scientific). Both Tb‐donor and QD‐acceptor photoluminescence (PL) intensities (in a time gate from 0.1 to 0.9 ms after pulsed excitation at 337 nm) were measured and the QD/Tb intensity ratios (*FRET‐ratio*) were used to record sEGFR concentration‐dependent immunoassay calibration curves. Increasing concentrations of sEGFR resulted in the formation of increasing amounts of Tb‐NB1‐EGFR‐NB2‐QD FRET sandwich complexes and a concomitant increase of the *FRET‐ratio*, which levelled off into saturation at higher sEGFR concentrations (Figure 1B).

**Figure 1:**
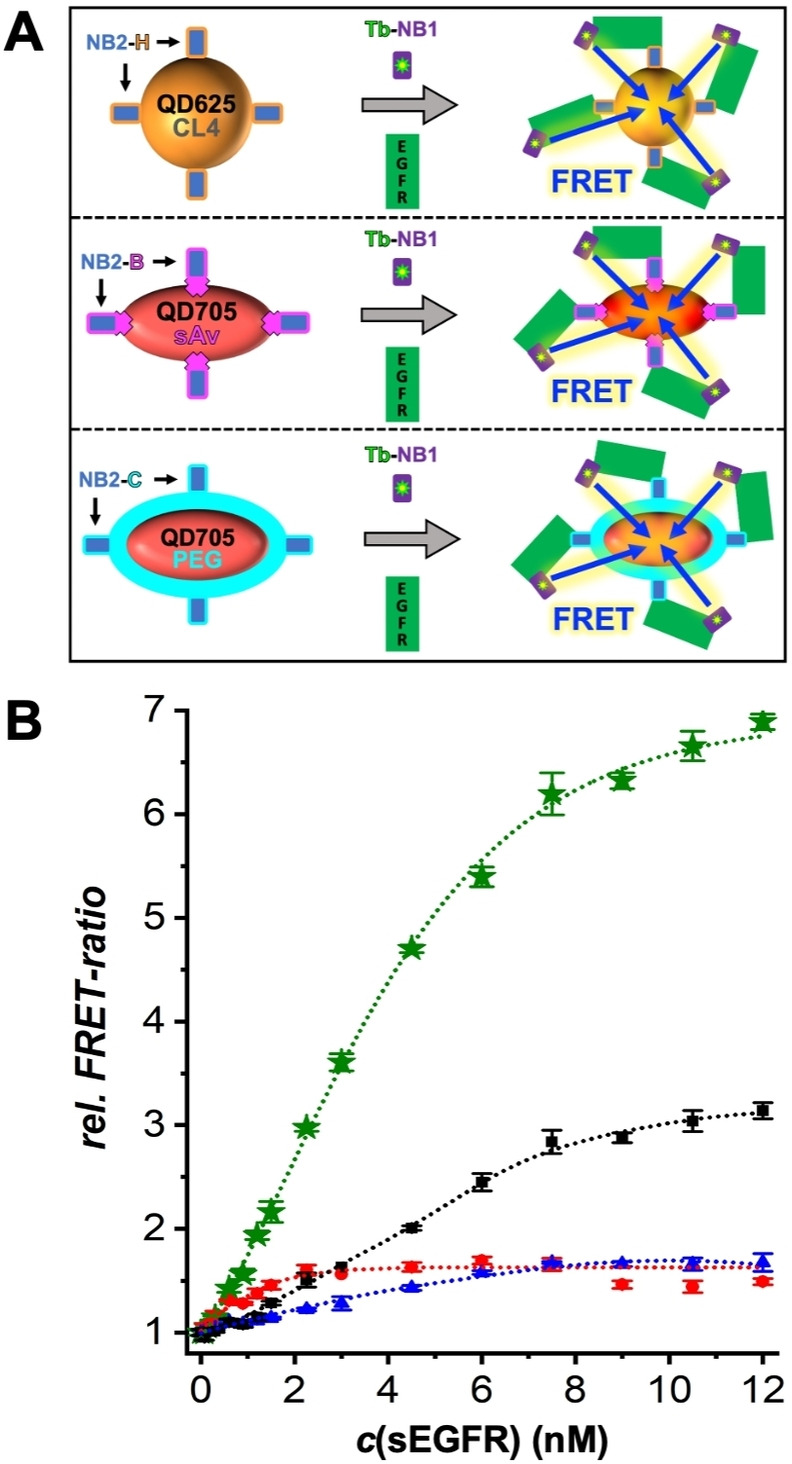
A) Principle of the NB‐based Tb‐to‐QD FRET sandwich immunoassays (see Table 1 for explanation of abbreviations). Mixing of NB‐QD acceptor conjugates (left side from top to bottom: NB2‐H‐QD625‐CL4, NB2‐B‐QD705‐sAv, and NB2‐C‐QD705‐PEG) with Tb‐NB1 donor conjugates and sEGFR (gray arrows in the center) resulted in the formation of immunological sandwich complexes with close Tb‐QD proximity for FRET (right side). Because the sEGFR is a Fc‐chimera homodimer and NB1 and NB2 bind to different epitopes of each monomer, different donor‐acceptor distances are possible, resulting in a mixture of high, low, and no FRET. The different sEGFR conformations and NB binding sites are shown in Supporting Figure S1. B) EGFR sandwich FRET immunoassay calibration curves (*rel. FRET‐ratio* is the *FRET‐ratio* normalized to the blank sample) using Tb‐NB1 as donor conjugates and NB2‐H‐QD625‐CL4 (1.5 nM—blue), NB2‐B‐QD705‐sAv (1.5 nM—black; 3.0 nM—green), and NB2‐C‐QD705‐PEG (1.5 nM—red) as acceptor conjugates. Data points represent three (10 for the blank samples without sEGFR) independent measurements. Error bars represent standard deviations. The sEGFR concentrations (0, 0.075, 0.15, 0.30, 0.60, 0.90, 1.20, 1.50, 2.25, 3.0, 4.5, 6.0, 7.5, 9.0, 10.5, and 12.0 nM) are those in the 50 μL sample (they are 3 times lower in the total 150 μL assay volume).

The typical shape of sandwich assay calibration curves (increase followed by saturation) was caused by saturated binding of Tb‐NB1 to sEGFR. The assays contained 50 μL of sample solution with different concentrations of sEGFR, 50 μL of NB2‐QD solution (1.5 nM or 3.0 nM QD), and 50 μL of Tb‐NB1 solution (6 nM NB1). The expected Tb‐NB1 saturation at ≈6 nM EGFR (when each sEGFR binds one Tb‐NB1) was confirmed for the assays containing NB2‐H‐QD625‐CL4 and NB2‐B‐QD705‐sAv. Considering that the same Tb‐NB1 conjugates were used for all experiments, saturation at ≈2 nM sEGFR for the assays containing NB2‐C‐QD705‐PEG must have been caused by limited binding of sEGFR to NB2‐QD. Thus, only ≈1.3 NBs (2 nM sEGFR/1.5 nM QD) were accessible on the QDs, which could have been caused by a low labelling ratio or by steric hindrance due to too dense labeling of NBs on the QD. This result confirmed the disadvantages of the Cys‐tag bioconjugation (vide supra), which requires bioconjugation at many different molar ratios to obtain optimized bioconjugates. Despite the differences in saturation, which resulted in a narrower dynamic concentration range for the NB2‐C‐QD705‐PEG (≈0.5 nM to ≈2 nM) compared to the NB2‐H‐QD625‐CL4 (≈0.8 nM to ≈8 nM) and NB2‐B‐QD705‐sAv (≈0.7 nM to ≈12 nM) conjugates, the assays were functional for all three NB‐QD conjugation strategies with similar limits of detection (LODs, 3 standard deviations above the zero concentration value) of 0.5±0.2 nM (NB2‐C‐QD705‐PEG), 0.7±0.2 nM (NB2‐B‐QD705‐sAv), and 0.8±0.2 nM (NB2‐H‐QD625‐CL4) sEGFR (Supporting Figure S4). The sensitivity (slope of the linear part of the assay calibration curve) was approximately tripled from 0.33±0.01 nM^−1^ to 1.00±0.03 nM^−1^ by increasing the NB2‐B‐QD705‐sAv concentration from 1.5 nM to 3 nM (green curve in Figure 1B), which also resulted in a lower LOD (0.20±0.05 nM, Supporting Figure S4) that was similar to NB‐based Tb‐QD FRET assays using different QDs and functionalization strategies.[^27^, ^29^]

The benefits of versatile and rapid NB‐QD bioconjugation and rapid and simple bioassays come with the drawback of relatively high LODs for quantifying sEGFR compared to other rapid FRET assays (using antibodies)^[29]^ or to the clinical cut‐off levels (threshold between normal and abnormal concentrations) of sEGFR (≈45 ng mL^−1^ or ≈0.22 nM).^[34]^ Therefore, we sought for exploiting the distinct properties of His_6_‐tagged NBs to develop a new FRET assay concept that can combine both simplification and higher sensitivity. In this assay design, Tb‐NB1‐H was noncovalently, and as such reversibly, attached to the surface of QD625‐CL4 via His_6_ self‐assembly, which led to Tb‐to‐QD FRET. Considering that both QD and sEGFR are significantly larger than NB, we hypothesized that this FRET signal can be disrupted by the release of Tb‐NB1‐H from QD625‐CL4 when NB1 specifically binds to sEGFR (Figure 2A). Despite that fact that the NB1‐sEGFR binding site is at the opposite end of the His_6_ tag (non‐competitive binding), the small size of the NB could possibly be sufficient, such that steric hindrance would lead to a successful displacement. Both His_6_ self‐assembly to QDs (*K*_D_≈1 nM)^[41]^ and binding of NB1 to EGFR (*K*_D_≈0.6 nM, Supporting Table S1) are relatively strong, such that the NB displacement assay would provide the possibility to detect FRET signal changes at low target concentrations, while at the same time requiring only a single NB (labelled with Tb) and unconjugated QDs within an assay test kit (which could be very beneficial for long‐term storage).

**Figure 2:**
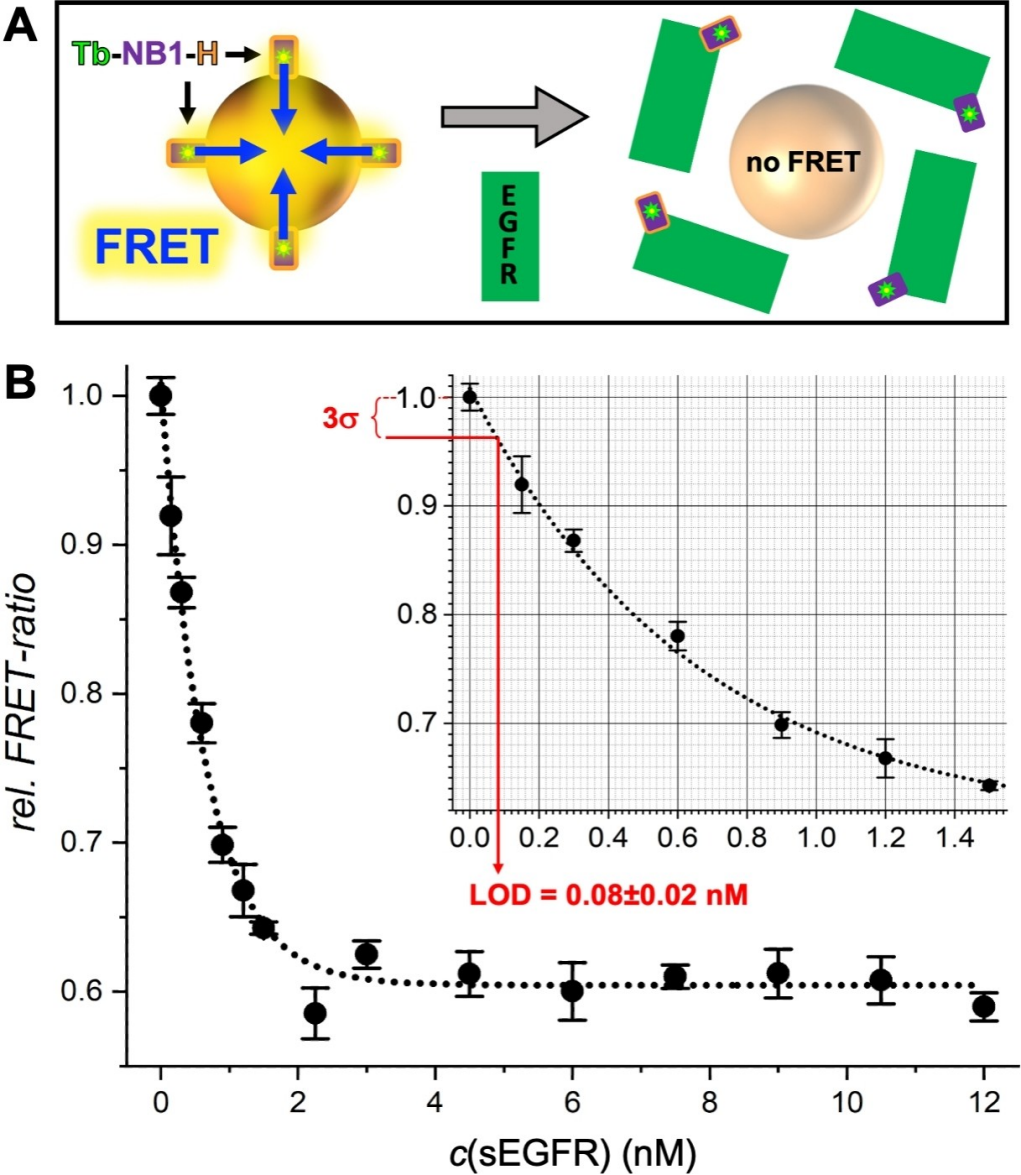
A) Principle of Tb‐to‐QD FRET NB displacement immunoassays. Mixing of Tb‐NB1‐H donor conjugate and QD625‐CL4 acceptor results in Tb‐to‐QD FRET (left). The addition of sEGFR (gray arrow in the center) leads to a displacement of Tb‐NB1‐H from the QD surface to sEGFR and disruption of FRET (right). B) NB displacement FRET immunoassay calibration curves with an LOD (3 standard deviations below the zero concentration value – see inset) of 0.08±0.02 nM (16±4 ng mL^−1^) sEGFR. Data points represent three (10 for the blank samples without sEGFR) independent measurements. Error bars represent standard deviations (σ). The EGFR concentrations are those in the 50 μL sample.

To experimentally demonstrate our hypothesis and for a direct comparison of the NB displacement FRET assay to the sandwich FRET assay, we started by simply replacing Tb‐NB1 with Tb‐NB1‐H in the sEGFR assay that used NB2‐H‐QD625‐CL4 as acceptor conjugates. Whereas the *FRET‐ratio* in the sandwich assay curve increased with increasing sEGFR concentration, it decreased for the NB displacement assay, clearly showing the inverse principle, i.e., high *FRET‐ratio* for Tb‐NB1‐H without sEGFR and low *FRET‐ratio* for the bound Tb‐NB1‐H‐EGFR complex. When NB2‐H‐QD625‐CL4 was replaced by unconjugated QD625‐CL4 (at otherwise identical assay conditions), the *FRET‐ratio* decrease was even stronger (Supporting Figures S5 to S7).

Knowing that the NB displacement sensing principle was functional with Tb‐NB1‐H and QD625‐CL4 as only assay components, we evaluated the assay performance at different Tb‐NB1‐H and QD625‐CL4 concentrations and with a focus on the subnanomolar concentration range to determine the LOD (Figure 2B). In contrast to the sandwich immunoassays, for which an increase in NB2‐QD concentration resulted in a better assay performance, the NB displacement assay performed significantly better at lower QD concentrations, most probably because of lower background signals that also resulted in lower standard deviations of the *FRET‐ratio*. The QD625‐CL concentration could be decreased 30‐fold from 1.5 nM to 0.05 nM, which presented another advantage concerning costs and efficient use of materials. In addition, by reducing the Tb‐NB1‐H concentration by 50 % from 6 nM to 3 nM, we could approximately triple the sensitivity from −0.133±0.017 nM^−1^ to −0.375±0.075 nM^−1^, as measured by the slope of the assay curve for low concentrations of sEGFR (Supporting Figure S8). Importantly, the LOD could be decreased to 80±20 pM (16±4 ng mL^−1^) sEGFR. This presented a 10‐fold improvement compared to the sandwich immunoassay using NB2‐H‐QD625‐CL4. The LOD is in the same range as antibody‐based sandwich assays,^[29]^ and 3‐fold lower than the clinical cut‐off level of 45 ng mL^−1^. Notably, all these benefits were accomplished by a significant simplification of assay production and assay format.

While the proof‐of‐concept of the novel NB displacement assay was clearly demonstrated, actual application in immunoassays for different targets requires the investigation of non‐specific binding, implementation for different relevant biomarkers and different NBs, and target specificity. We first evaluated the NB displacement assay for the two biomarkers sEGFR and sEGFRvIII in serum containing samples, for which the many serum components can potentially cause significant non‐specific binding and thus reduce the assay performance. Notably, TG detection from 0.1 to 0.9 ms after pulsed excitation at 337 nm efficiently suppressed both directly excited QD PL and sample autofluorescence, such that the serum components did not result in any additional background signal (Supporting Figure S9). Considering that commercial ELISA kits for human sEGFR use at least 10‐fold dilution of serum samples,^[42]^ we investigated samples containing 5 to 30 vol % of fetal bovine serum (FBS). Although the assay performance decreased with increasing serum fractions, all FRET‐ratios showed a clear target concentration dependence for both sEGFR and sEGFRvIII (Supporting Figure S10). We selected 5‐fold dilution of serum samples (i.e., 20 vol %) for a direct comparison of the assay performance in buffer and serum for both sEGFR and sEGFRvIII (Figure 3). While the assay calibration curves for serum samples showed slightly lower sensitivity and overall reduction of the *FRET‐ratio* change, both sEGFR and sEGFRvIII could be quantified in the same concentration range as for the buffer samples.

**Figure 3:**
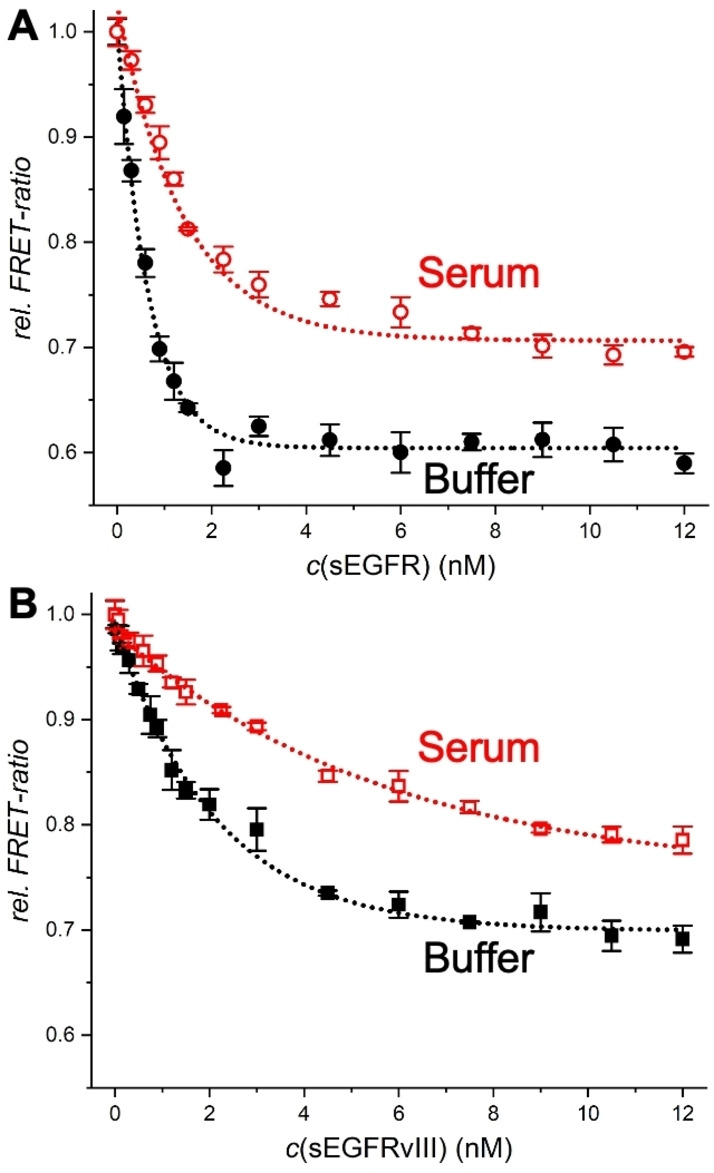
Comparison of NB displacement immunoassays for sEGFR (A) and sEGFRvIII (B) in serum (20 vol %) and buffer. Data points represent three (10 for the blank samples without sEGFR) independent measurements. Error bars represent standard deviations (σ). The EGFR concentrations are those in the 50 μL sample.

Interestingly, sEGFRvIII quantification (Figure 3B) is less sensitive than sEGFR quantification (Figure 3A), which can be explained by the reduced domain **II** of sEGFRvIII (only residues 273–311 are retained compared to sEGFR)^[37]^ and the binding of NB1 in the cleft formed between domains **II** and **III**. Thus, despite the independent binding of NB1 to the target and QD (EGFR binding site at the opposite end of the His_6_ tag), the NB displacement assay performance was dependent on the NB‐target binding position and affinity. To further investigate the influence of NB‐target binding, we used NB2 instead of NB1. Although the NB2‐based displacement assay was functional, the assay curves showed significantly less sensitivity for sEGFR detection and serum fractions of more than 10 vol % resulted in only very weak sEGFR concentration dependence (Supporting Figure S11). Because the affinities of NB1 (≈0.6 nM, Supporting Table S1) and NB2 (≈0.7 nM, Supporting Table S1) for sEGFR are very similar, the different displacement behavior can only be explained by the different binding sites (NB2 binds to domains **I** and **II**). The weak displacement of NB2 from the QDs also provided an explanation for the lowest sensitivity (smallest slope of calibration curve) of the NB2‐H‐QD625‐CL4 (compared to NB2‐B‐QD705‐sAv and NB2‐C‐QD705‐PEG) sandwich immunoassays (Figure 1), despite the fact that the Tb‐QD distance was shortest for the CL4 coated QDs. The NB2‐H‐QD625‐CL4 sandwich assay was most probably driven by a combination of displacement and sandwich formation, with a slight advantage of sandwich formation and thus, an increasing *FRET‐ratio* with increasing sEGFR concentration. Tuning the displacement efficiency by the NB‐target binding sites presents an important lever for optimizing NB‐displacement assays and an extensive investigation with a large library of NBs and targets would be highly interesting to fully understand the potential of this novel type of assay.

Finally, we evaluated the specificity of the NB‐displacement assays for sEGFR and the possibility of shortening the assay incubation time. Both NB1 and NB2 based assays did not show any significant *FRET‐ratio* changes when the soluble epidermal growth factor receptor 2 (sHER2) was used instead of sEGFR (Supporting Figure S12). In addition, we showed the potential for further assay simplification by reducing the incubation time to only 15 to 30 minutes, which was found to be sufficient for sEGFR quantification (Supporting Figure S13). Again, a more detailed study with many different targets and NBs would be necessary to fully assess specificity and simplicity optimization possibilities.

In conclusion, we developed a nanobody‐tag toolkit for simple, versatile, and efficient bioconjugation to QDs with different surface coatings, sizes, and PL colors. The immunotargeting functionality of the NB‐QD conjugates was demonstrated on homogeneous TG‐FRET immunoassays against sEGFR, using two different, non‐competing EGFR‐specific NBs (NB1 functionalized with Tb and NB2‐tag attached to QDs). Despite the differences of QD sizes and coatings and NB orientations on the QD surfaces, NB1‐EGFR‐NB2 sandwich complexes were formed for all NB‐QD conjugates, which resulted in an sEGFR concentration‐dependent increase of Tb‐to‐QD FRET with subnanomolar LODs in low‐volume (50 μL) samples and the best assay performance when using biotin‐tagged NBs and sAv‐coated QDs. QD‐bioconjugation with His_6_ and biotin tagged NBs was significantly faster, simpler, and easier to quantify compared to Cys tagged NBs. A significantly simplified immunoassays format combined with improved analytical performance was realized by exploiting the displacement of His_6_‐tagged Tb‐NB1 from the QD surface. Despite the independent (non‐competitive) binding of His_6_ to the QD surface and Tb‐NB1 to sEGFR, NB‐displacement was sEGFR concentration‐dependent. The displacement mechanism was further studied using a different target (sEGFRvIII) and a different NB (NB2), which showed that modification of the NB‐target binding site can be used for tuning the displacement efficiency. The rapid, wash‐free, and simple assay format (only on Tb‐NB conjugate+unconjugated QDs) required 30‐fold less QDs and afforded an LOD of 80±20 pM (16±4 ng mL^−1^), which was 3‐fold below the clinical cut‐off level of sEGFR. The relevance of the NB‐displacement assay for clinical diagnostics was further demonstrated by quantifying both sEGFR and sEGFRvIII in serum samples. Mix‐and‐measure NB‐on‐QD displacement FRET assays can significantly reduce assay development, production, and material costs, improve QD storage conditions in assay kits, and provide clinically relevant analytical performance for biomarker quantification, all of which are highly important benefits for translating QD‐based biosensors into daily use in bioanalytical research and clinical diagnostics.

## Conflict of interest

The authors declare no conflict of interest.

## Supporting information

As a service to our authors and readers, this journal provides supporting information supplied by the authors. Such materials are peer reviewed and may be re‐organized for online delivery, but are not copy‐edited or typeset. Technical support issues arising from supporting information (other than missing files) should be addressed to the authors.

Supporting Information

## Data Availability

The data that support the findings of this study are available in the supplementary material of this article.
