# Supplementary material for: A Nanobody‐on‐Quantum Dot Displacement Assay for Rapid and Sensitive Quantification of the Epidermal Growth Factor Receptor (EGFR)
Source: Angew Chem Int Ed Engl. 2022 Jul 8;61(33):e202207797. doi: 10.1002/anie.202207797 (PMC9542526; doi:10.1002/anie.202207797)
Supplement: Supplementary file 1 — Supporting Information [file ANIE-61-0-s001.pdf]

Angewandte Chemie
Eine Zeitschrift der Gesellschaft Deutscher Chemiker

Supporting Information

A Nanobody-on-Quantum Dot Displacement Assay for Rapid and Sensitive Quantification of the Epidermal Growth Factor Receptor (EGFR)

R. Su, Y.-T. Wu, S. Doulkeridou, X. Qiu, T. J. Sørensen, K. Susumu, I. L. Medintz, P. M. P. van Bergen en Henegouwen*, N. Hildebrandt**

Table of Contents

Materials..…………………………………………………………………………….………………S3

Figure S1. Schematic presentation of EGFR conformations and NB binding….……………............…S4

Figure S2: Binding of NBs to A431 cells……………………………………………………………S4

Table S1: Binding data from binding assays in Figure S2………………………………………......S4

Figure S3. Emission and absorption spectra of Tb, QD625, and QD705………………………………S5

QD-NB conjugation……………………………………………………………………...……S5

Tb-NB conjugation………………...…………………………………………………………………S6

Optical characterization of FRET pairs……………………………………………….………………S6

FRET immunoassays against EGFR………………………………………………………………..S7

Figure S4. PL decay curves and assay calibration curves for sandwich immunoassays………….........S8

Figure S5. Initial NB displacement FRET immunoassay calibration curves…………………………..…S9

Figure S6. PL decay curves for NB displacement immunoassays…………………………….………...S10

Figure S7. PL decay curves for NB displacement immunoassays……………………………………....S11

Figure S8. Final NB displacement FRET immunoassay calibration curves……………………………S11

Figure S9. Autofluorescence evaluation……………………………………………………………..S12

Figure S10. Influence of non-specific binding serum components……………………………………...S13

Figure S11. Influence of different NBs………………………………………................................S13

Figure S12. Specificity evaluation of NB displacement FRET assay…………………….......................S14

Figure S13. Incubation time dependence of NB displacement FRET assays ……………………...........S14

References……………………………………...………………………………………….....S15

Materials.

Recombinant human EGFR Fc chimera (#344-ER-050) and HER2 Fc chimera (#1129-ER-050) dimers (MW: ~190 kDa for EGFR dimer and ~195 kDa for HER2 dimer) were purchased from R&D system. Human EGFRvIII, Fc Tag (EGI-H5255, ACRObiosystems) was purchased at Fisher Scientific. 705nm emitting Qdots ITK Streptavidin (for size estimation: sAv has a size of 5.4 nm x 5.8 nm x 4.8 nm in the solid state)[1] Conjugate Kit (sAvQD705 - core-shell ellipsoid size of around 6 nm × 12 nm minimum/maximum axes, as measured with TEM by the manufacturer; hydrodynamic diameter including sAv of ~18.5 nm as measured by size exclusion chromatography on HPLC by the manufacturer and size determined via retention time relative to a standard curve of proteins; circa 6 to 10 sAv per QD as given by the provider and estimated in biotin binding experiments), 705nm emitting ITK Qdots with amino-PEG (QD705 - core-shell ellipsoid size of around 6 nm × 12 nm minimum/maximum axes, as measured with TEM by the manufacturer; hydrodynamic diameter including amino-PEG of ~20.5 nm as measured by size exclusion chromatography on HPLC by the manufacturer and size determined via retention time relative to a standard curve of proteins) and 625nm emitting organic Qdots (QD625, core-shell diameter of ~9.2 nm)[2] were purchased from Thermo Fisher. Lumi4-Tb complexes functionalized with NHS were provided by Lumiphore Inc.

Trizma hydrochloride, phosphate buffered saline (1×PBS), hydrochloric acid (HCl), sodium hydroxide (NaOH), tris(2-carboxyethyl)phospine hydrochloride (TCEP), N,N-dimethylformamide (DMF), sodium tetraborate decahydrate ($Na_2B_4O_7 \cdot 10H_2O$), sodium bicarbonate ($NaHCO_3$), bovine serum albumin (BSA) fetal bovine serum (FBS), and sulfo-EMCS crosslinker were purchased from Sigma-Aldrich. Sodium chloride (NaCl) was purchased from Duchefa. All chemicals were used as received. High-quality Milli-Q water with a resistivity of 18.2 MΩ cm was used for preparing solutions.

Selection of NBs was previously described and included EgB4 (NB2), a NB binding to both domain **I** and **II** of sEGFR,[3,4] and EgA1 (NB1), a NB binding to both domain **II** and **III**.[3,5] Conjugation of the NBs to the different tags did not affect their affinity for EGFR, as measured by binding to A431 cells (**Figure S2** and **Table S1**).

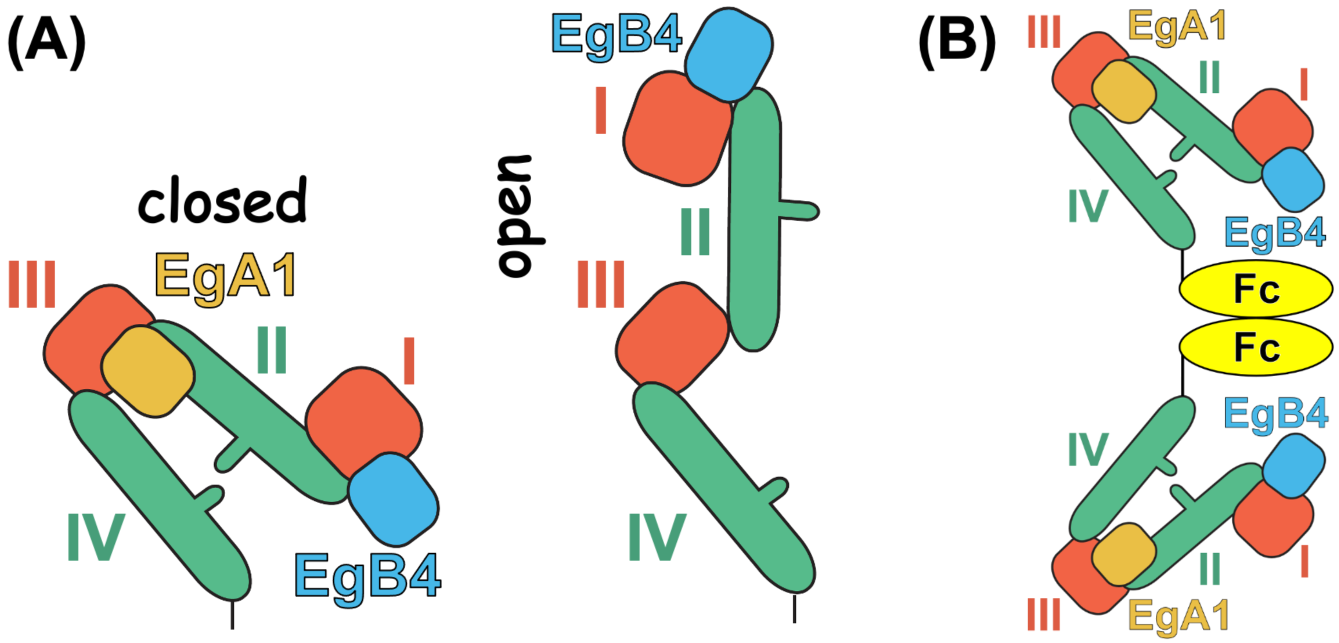

Figure S1. Schematic presentation of EGFR conformations and binding of EgA1 (NB1) and EgB4 (NB2). **(A)** The four ectodomains of EGFR with bound EgA1 and EgB4. In the open form (right), only EgB4 can bind. EgA1 binds in the cleft formed between domains **II** and **III** and keeps EGFR in that closed form (left).[5] In the presence of both EgA1 and EgB4, the equilibrium between the two conformations is most probably shifted to the closed form. **(B)** Possible EGFR Fc-homodimer conformation when both EgA1 and EgB4 bind to EGFR. The two EgA1 and two EgB4 per Fc-homodimer, the different possible conformations, and the flexibility in the Fc fusion sites result in many possible donor-acceptor distances and thus, a donor-acceptor distance distribution.

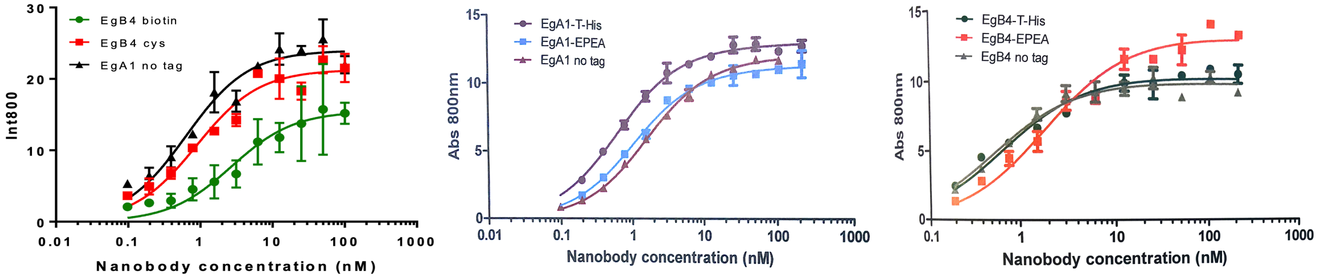

Figure S2. Results of binding assays performed with different NBs on A431 cells.

Table S1: Results from the different binding assays shown in Figure S2.

	EgB4 biotin	EgB4 Cys	EgB4-T-His	EgB4-EPEA	EgB4 no tag	EgA1-T-His	EgA1-EPEA	EgA1 no tag	EgA1 no tag
B_{max}	15.5	21.3	10.2	13.1	9.9	12.4	10.8	11.5	24
k_D	2.7 nM	0.9 nM	0.7 nM	1.9 nM	0.6 nM	0.6 nM	1.0 nM	1.5 nM	0.6 nM

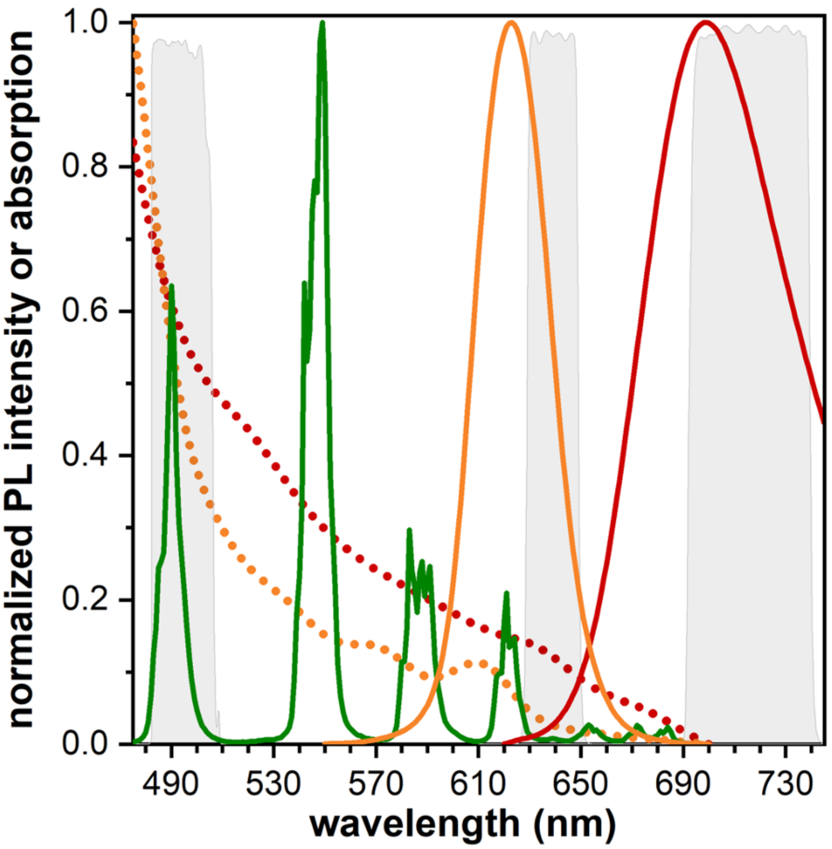

Figure S3. PL emission spectra of Tb (green), QD625 (orange), and QD705 (red) and spectral bandwidths of the filters (gray areas in the background) that were used for Tb and QD detection in the immunoassays. QD absorption spectra (dotted lines) showed good spectral overlap with the Tb PL (Förster distances: R_0(Tb-QD625) = 9.7±0.5 nm; R_0(Tb-QD705) = 10.4±0.5 nm). Excitation at 337 nm was close to the maximum absorption (~339 nm) of Tb (not shown).

QD-NB conjugation.

Conjugation of EgB4-Cys (NB2-C) to amino-PEG coated QD705 was performed using sulfo-EMCS crosslinkers. To receive maleimide-reactive QD705, a >50,000-fold molar excess of sulfo-EMCS was mixed with QD705 for 1h with 30 rpm at room temperature. Maleimide-activated QDs were purified using 100 kDa MWCO spin column from Millipore (Billerica, MA, USA) by washing three times with 1×PBS buffer (pH 7.4) to remove excess crosslinker. Disulfide bonds of EgB4 were reduced to sulfhydryls with 5 mM TCEP by mixing for 30 min with 30 rpm at room temperature without further purification. For final conjugation, both solutions (43µl of 130.9 µM EgB4 + 100µl of 0.64 µM QD705) were mixed and incubated for 6 h with 30 rpm at room temperature in the dark. Unbound EgB4 were separated by 100 kDa MWCO spin column by washing four times with 100 mM sodium tetraborate buffer (pH 8.4). Purified conjugates were centrifuged at 4000g and supernatants were stored at 4°C. QD concentrations were determined by absorbance measurements using a molar extinction coefficient of 8.3×10^6 M^{-1} cm^{-1} (at

405 nm) for QD705 as provided by the manufacturer. EgB4 were quantified by absorbance measurement at 280 nm using an extinction coefficient of 35,540 M^{-1} cm^{-1}. The labeling ratio (~28 NB/QD) was determined by linear combination of the respective absorbance values of QD705 and EgB4 within the EgB4-Cys-QD705 conjugates. Considering the more than 200-fold difference in extinction coefficients between QD705 and NB2 at 280 nm, quantification of the labeling ratio by absorption measurements is very inaccurate and can only provide a rough estimation. Therefore, the actual labeling ratio is somewhere in between zero NB2 per QD and 90 NB per QD (which was the molar ratio in the labeling procedure). QD625s were functionalized with CL4 compact ligands as described previously.[6] Conjugation of EgB4-His_6 (NB2-H) to QD625 was performed in a molar ratio of 20:1 (EgB4-His_6 per QD) and mixed for 30 min. For best reproducibility, the conjugates were freshly prepared before FRET immunoassays. However, we also performed functional assays with NB-QD conjugates that had been stored for several months with no significant differences (not shown). QD concentrations were determined by absorbance measurements using a molar extinction coefficient of 9.9×10^6 M^{-1} cm^{-1} (at 405 nm) for QD625 as provided by the manufacturer. EgB4 was quantified by absorbance measurement at 280 nm using an extinction coefficient of 37,520 M^{-1} cm^{-1}. Taking into account the very efficient self-assembly of His_6-tagged biomolecules to QDs, the molar ratio can be considered as the actual labeling ratio (~20 NB/QD). Conjugation of EgB4-biotin (NB2-B) to sAv-QD705 was performed in a molar ratio of 20:1 (EgB4-biotin per QD) and mixed for 30 min. For best reproducibility, the conjugates were freshly prepared before FRET immunoassays. However, we also performed functional assays with NB-QD conjugates that had been stored for several months. EgB4 was quantified by absorbance measurement at 280 nm using an extinction coefficient of 38,060 M^{-1} cm^{-1}. Taking into account the very efficient biotin-sAv binding, the molar ratio can be considered as the actual labeling ratio (~20 NB/QD).

Tb-NB conjugation.

8 mM Lumi4-Tb-NHS was dissolved in anhydrous DMF and mixed (reaction ratio of Tb/AB = 3) with EgA1 or EgA1-His_6 (NB1 or NB1-H) in 100 mM carbonate buffer (pH 9.0). The mixture was incubated for 2 h with 25 rpm at room temperature. For Tb-conjugate purification, the samples were washed four to six times with 100 mM TRIS-HCl (pH 7.4) using 3 kDa MWCO Amicon spin column at 4000g. Tb concentration was determined by absorbance measurements at 340 nm using a molar absorptivity of 26,000 M^{-1} cm^{-1} as provided by the manufacturer. NBs were quantified by absorbance measurements at 280 nm using an extinction coefficient of 34,510 M^{-1} cm^{-1} (EgA1) and 38,880 M^{-1} cm^{-1} (EgA1-His_6). The conjugation ratios were determined by a linear combination of the respective absorbance values of Tb and NBs within the Tb-NB conjugate absorbance. For both Tb-EgA1 and Tb-EgA1-His_6, the labeling ratios were 1.4±0.7 Tb/NB.

Optical characterization of FRET pairs.

Absorption spectra (Lambda 35 UV/Vis System, PerkinElmer) and emission spectra for Tb (FluoTime 300, PicoQuant) and QD (SAFAS) samples were recorded in TRIS-HCl (100 mM, pH 7.4) respectively.

FRET immunoassays against sEGFR.

Time-gated PL intensity measurements were acquired on a KRYPTOR compact PLUS clinical fluorescence plate reader (Thermo Fisher Scientific) in a time window from 0.1 ms to 0.9 ms after pulsed excitation at 337.1 nm using an integrated nitrogen laser operating at 20 Hz, 100 pulses. The PL decay curves were acquired directly from the FRET immunoassay samples on an time-resolved fluorescence plate reader (Edinburgh Instruments) using 4000 detection bins of 2 µs integration time and nitrogen laser (VSL 337 ND, SPECTRA Physics) excitation at 337.1 nm, 20 Hz. Optical bandpass filters (Delta and Semrock) for Tb donor and QD acceptor channel were 494±10 nm for Tb, 640±7 nm for QD625, and 716±20 nm for QD705.

Tb-EgA1 and EgB4-QD conjugates were each dissolved in 50 µL of 10 mM TRIS-HCl buffer (pH 7.4) containing 0.5% (*w/v*) BSA. For FRET sandwich immunoassay calibration curves against sEGFR, the overall measuring volume of 150 µL contained 100 µL of a constant assay solution (50 µL of 6 nM Tb-EgA1 and 50 µL of 1.5 nM or 3.0 nM EgB4-QD conjugate) and 50 µL of sEGFR sample solution (with different concentrations of EGFR in TRIS/BSA buffer). For NB displacement FRET immunoassay calibration curves against sEGFR and sEGFRvIII, the overall measuring volume of 150 µL contained 100 µL of a constant assay solution (50 µL of 6 nM or 3 nM Tb-EgA1 and 50 µL of 1.5 nM EgB4-QD625 or 1.5 nM or 0.15 nM unconjugated QD625) and 50 µL of sEGFR or sEGFRvIII sample solution (with different concentrations of sEGFR or sEGFRvIII in TRIS/BSA buffer or in TRIS/BSA buffer containing between 5 and 30% (*v/v*) FBS). Optical bandpass filters (Delta and Semrock) were 494±20 nm for Tb, 640±14 nm for QD625, and 716±40 nm for QD705. All FRET assays were measured in black 96-well microtiter plates with an optimal working volume of 150 µL. Each sample containing sEGFR was prepared three times and the samples without sEGFR were prepared 10 times. Each of the samples was measured independently, such that the data points consisted of three or ten independent measurements and standard deviations were used as errors. After sample preparation the microtiter plates were incubated for 180 min (for sandwich assays) or 120 min (for NB displacement assays) at 37° C before measurements on KRYPTOR compact PLUS and Edinburgh Instruments time-resolved fluorescence plate readers. NB displacement assays were also performed after different incubation times (15, 30, 45, 60, 120, and 180 min) and with sHER2 instead of sEGFR.

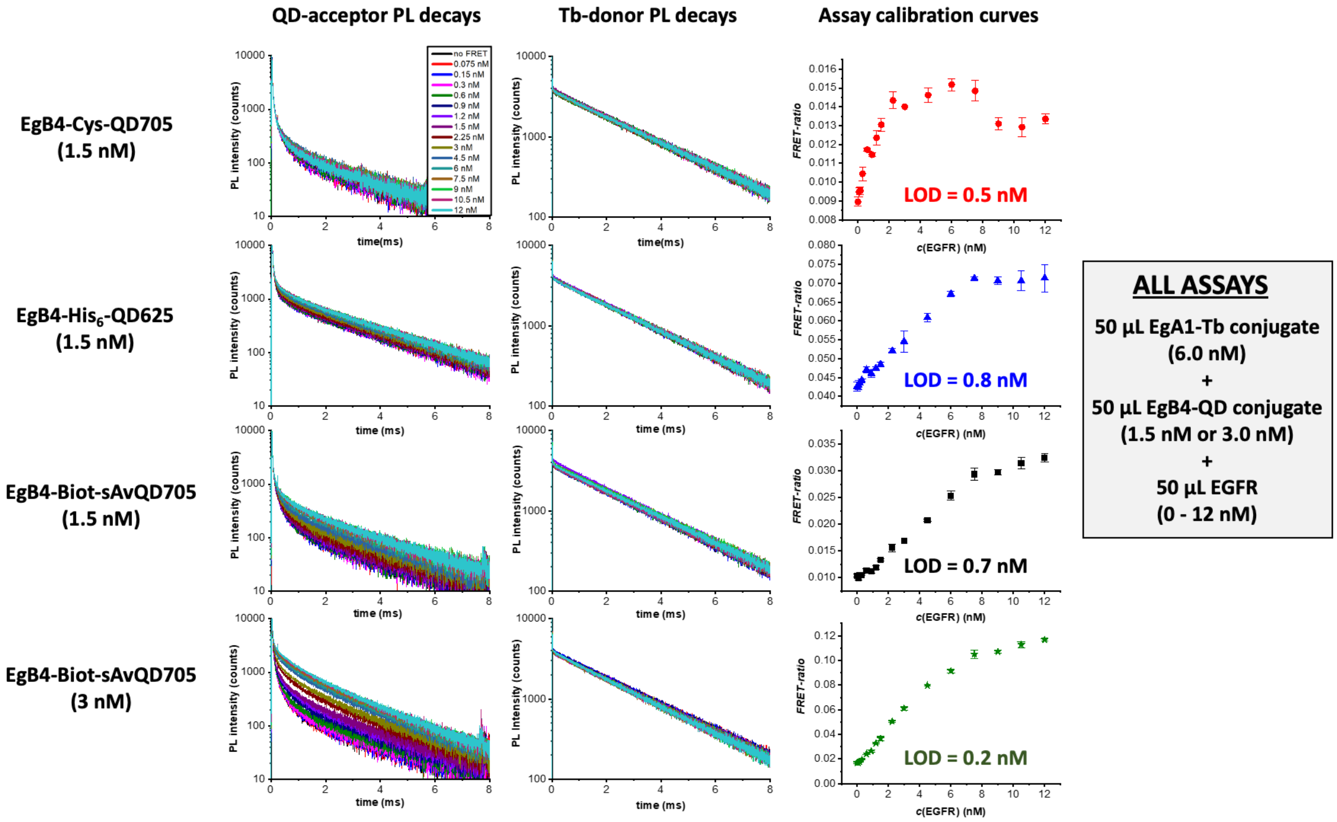

Figure S4. QD-acceptor (left) and Tb-donor (center) PL decay curves for sandwich immunoassays at different EGFR concentrations. QD-FRET-sensitization (increasing PL intensities with increasing EGFR concentrations) was strongest for EgB4-Biot-sAv-QD705 (and stronger for 3.0 nM compared to 1.5 nM QD concentration), followed by EgB4-His$_6$-QD625, and EgB4-Cys-QD705. Tb-FRET-quenching is much less intense (PL intensity differences are very weak and not well visible in the PL decay curves) because many Tb on the EgA1 will be too far away from the QD to engage in efficient FRET. Therefore, only the closest Tb will efficiently sensitize the QDs. PL decay curves were measured on a time-resolved fluorescence plate reader (Edinburgh Instruments), whereas the assay calibration curves (right) were measured on a KRYPTOR compact PLUS clinical diagnostic reader system (Thermo Fisher Scientific), which measures the PL intensities of QD and Tb in a time-gated detection window from 0.1 to 0.9 ms after pulsed excitation with a nitrogen laser at 337.1 nm and calculates the QD/Tb PL intensity ratio (FRET ratio) to perform ratiometric detection. LODs are the concentrations corresponding to the FRET ratio 3-times above standard deviation of the zero-concentration sample on the calibration curves.

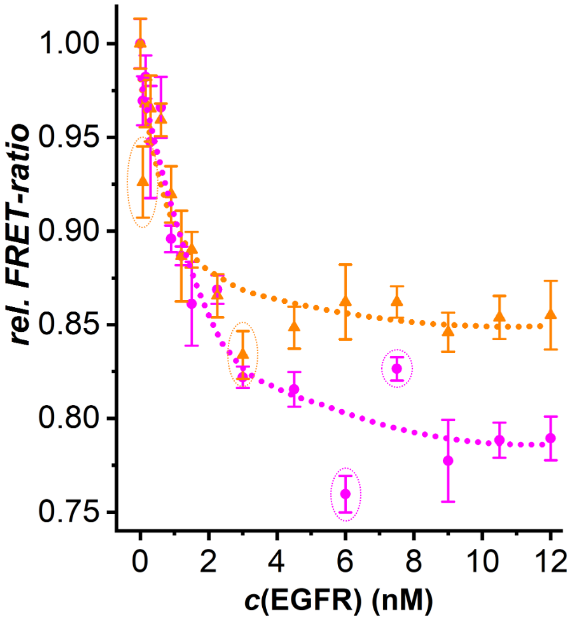

Figure S5. NB displacement FRET immunoassay calibration curves using Tb-EgA1-His_6 as donor NB conjugates and EgB4-His_6-QD625 (orange) or unconjugated QD625 (magenta) as acceptors. Note: The EGFR concentrations are those in the 50 µL sample (they are 3 times lower in the total 150 µL assay volume). Circled data points were considered as outliers. Whereas the FRET-ratio in the sandwich assay curve (blue assay curve in **Figure 1B** in the manuscript) increased linearly up to ~1.7 fold at ~8 nM sEGFR, it decreased very steeply to ~0.87-fold at ~4 nM sEGFR for the NB displacement assay (orange assay curve). When NB2-H-QD625-CL4 was replaced by unconjugated QD625-CL4, the FRET-ratio decrease was even stronger (magenta assay curve).

To better understand the differences shown in **Figure S5**, we took a closer look at the PL decay curves of Tb donors and QD acceptors (**Figures S6 and S7**). When both NBs (Tb-NB1-H and NB2-H) were attached to the QD, addition of sEGFR resulted in decreased intensity of the short QD PL decay (short distance), increased intensity of the medium QD PL decay (medium distance), and unchanged intensity of the long PL decay, which was caused by spectroscopic crosstalk of Tb PL into the QD detection channel (long distance without FRET). When only Tb-NB1-H was present, the short distance component decreased very similarly with increasing EGFR concentration (displacement of Tb-NB1-H from QD625-CL4) but the medium distance component increased almost two times less (Tb-NB1-H-EGFR-NB2-H-QD625-CL4 formation was not possible because NB2-H was not present in the assay). These different intensity changes of FRET at distinct distances showed that after displacement of Tb-NB1-H from QD625-C by sEGFR binding, the formation of sandwich complexes (with NB2-H still present on the QD) was still possible and that NB displacement was more efficient when only Tb-NB1-H was used in the assay.

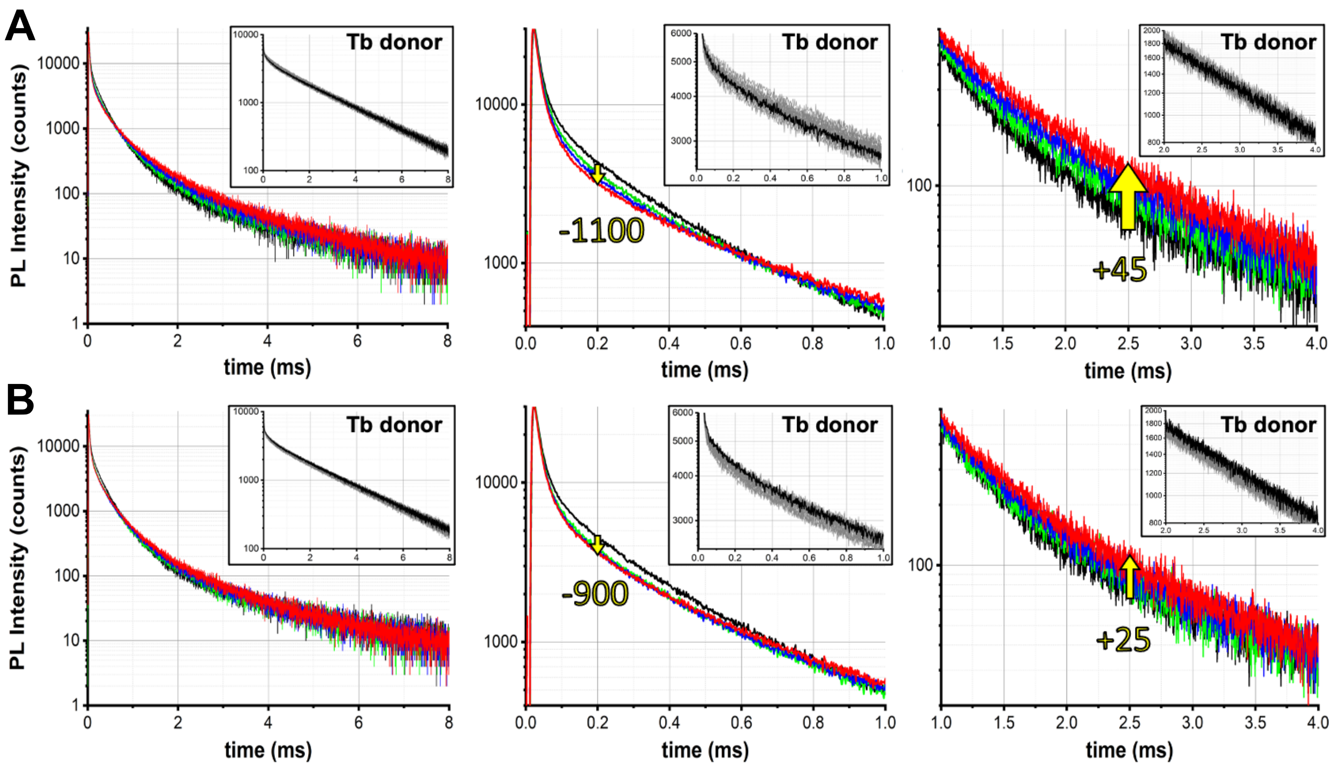

Figure S6. QD-acceptor and Tb-donor (insets) PL decay curves of the NB displacement EGFR immunoassays with Tb-EgA1-His6 and EgB4-His6-QD625 (**A**) and Tb-EgA1-His6 and unconjugated QD625 (**B**) with no EGFR (black curves) and 1.2 nM (green), 2.25 nM (blue), and 7.5 nM (red) EGFR (curves for all measured concentrations are shown in **Figure S7**). For the Tb PL curves all non-zero EGFR concentrations are shown in gray because there EGFR concentration dependent trend was much less pronounced. The graphs from left to right contain the same PL decay curves but on different time scales. (**A**) The short QD PL decay component (zoom in the center) decreases by ~1100 counts at 0.2 ms (lower fraction of Tb-EgA1-His6-QD at short Tb-QD distance) and the medium QD PL decay component (zoom on the right) increases by ~45 counts at 2.5 ms (higher fraction of Tb-EgA1-His6-EGFR-EgB4-His6-QD at slightly longer Tb-QD distance). At the same time, the short (and also slightly the medium) Tb PL decay component increases because overall there are more Tb-EgA1-His6 attached to the QD because there are 5 times less TbEgA1-His6 (6 nM) than EgB4-His6 (30 nM because there are 20 EgB4-His6 per 1.5 nM QD) in the assay. (**B**) The short QD PL decay components decrease very similarly by ~900 counts at 0.2 ms (lower fraction of Tb-EgA1-His6-QD at short Tb-QD distance) but the medium QD PL component increases much less by only ~25 counts at 2.5 ms (note that the overall counts in this time region are in the 50 to 150 counts region). At the same time, the short (and also slightly the medium) Tb PL decay component decreases because there is no second binding possibility (only on or off).

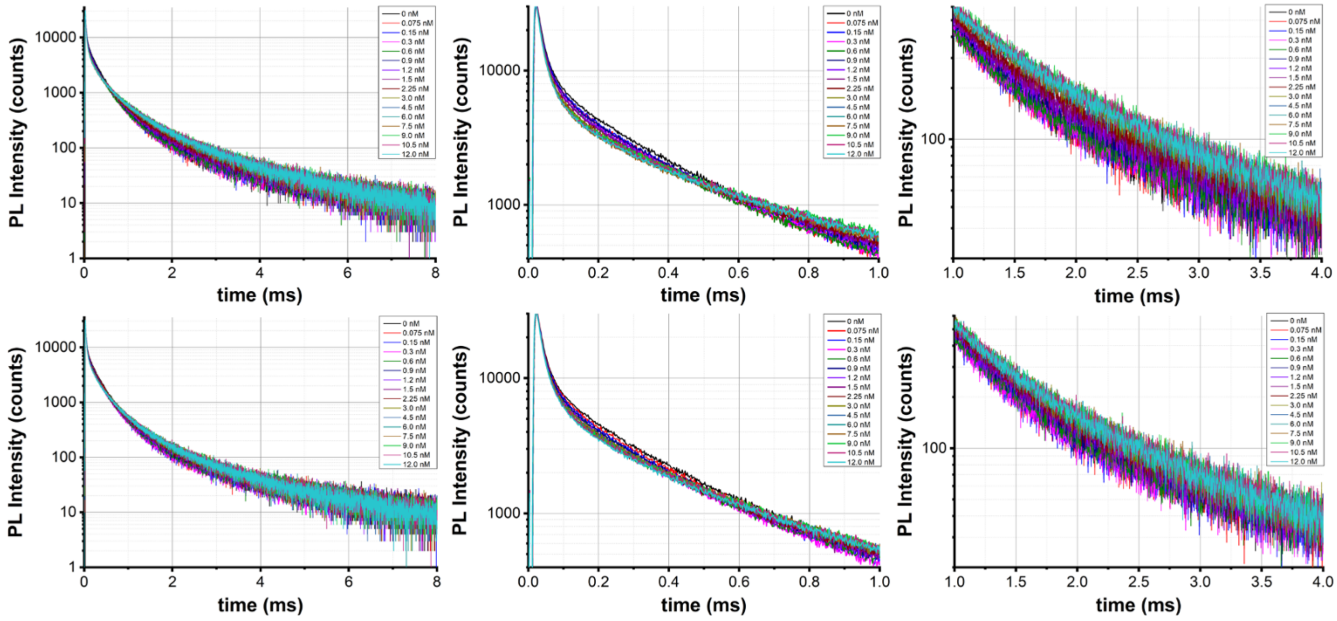

Figure S7. Same experiments as shown in **Figure S6** but with all measured concentrations.

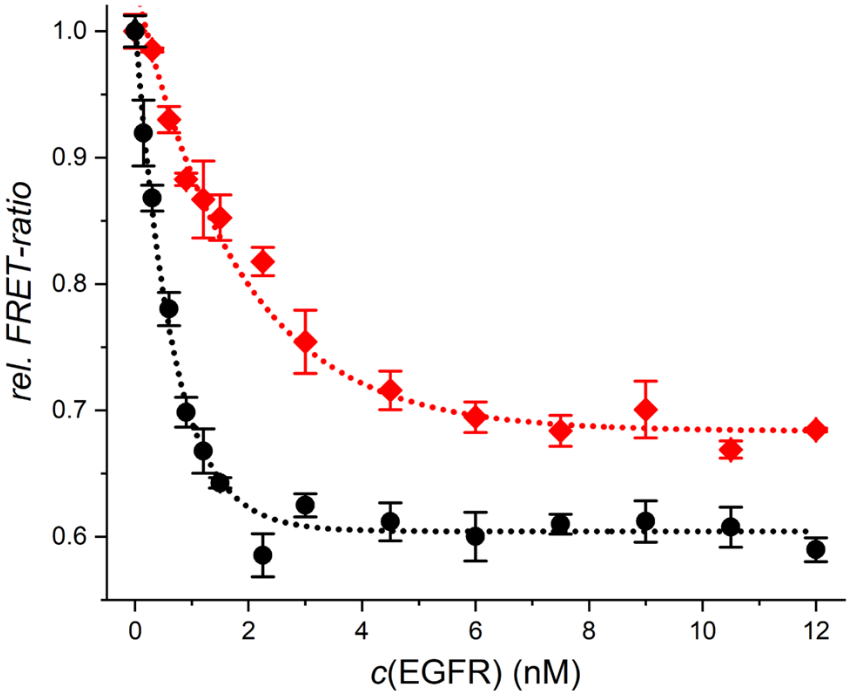

Figure S8. NB displacement FRET immunoassay calibration curves of assay mixtures containing 50 µL of different sEGFR concentrations (as given on the x axis), 50 µL of 0.05 nM QD625-CL4, and 50 µL of 6 nM (red diamonds) or 3 nM (black dots) of Tb-NH1-H.

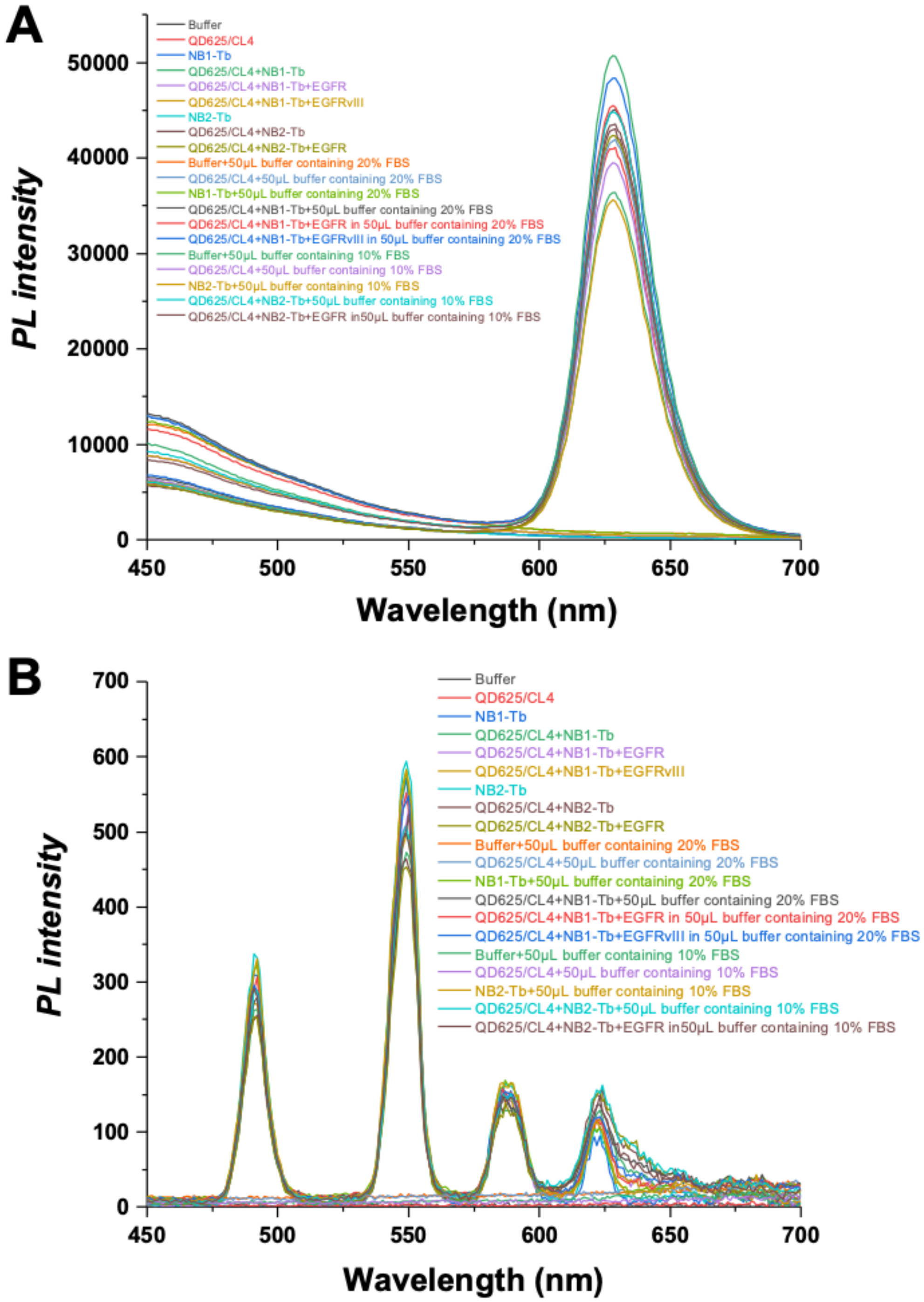

Figure S9. A: Steady-state detection with 337 nm excitation of different assay mixtures in the 450 to 700 nm range shows strong sample autofluorescence (~450 to 600 nm) and/or PL from directly excited QDs (~600 to 675 nm), whereas the Tb PL is negligible. **B:** TG detection (0.1 ms to 0.9 ms) with 337 nm excitation of the same samples completely suppresses the autofluorescence background and PL from directly excited QDs (both have PL lifetimes in the nano to microsecond range) and only long-lived Tb donor PL and sensitized long-lived QD PL becomes detectable.

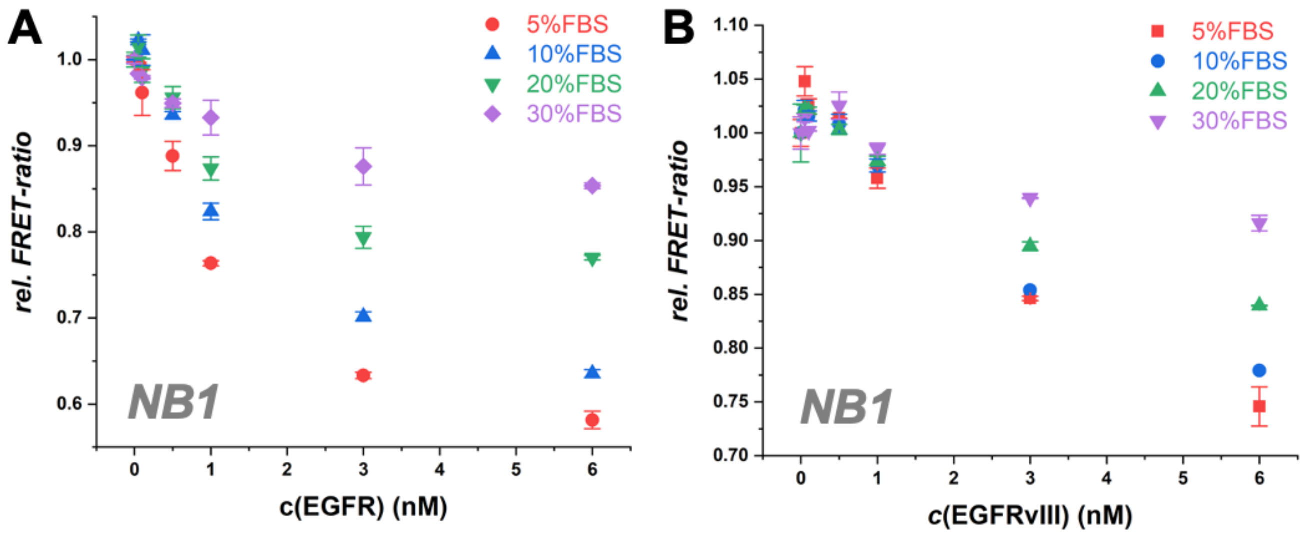

Figure S10. Influence of non-specific binding serum components. Relative FRET-ratios of the NB1+QD system against different sEGFR (**A**) or sEGFRvIII (**B**) concentrations (0.05, 0.1, 0.5, 1, 3, 6 nM respectively) in 50 µL buffer containing different amount of FBS (5%, 10%, 20%, 30%). For all measurements, incubation time was 90 min. Data points represent the measurement of three independent samples. Error bars represent standard deviations.

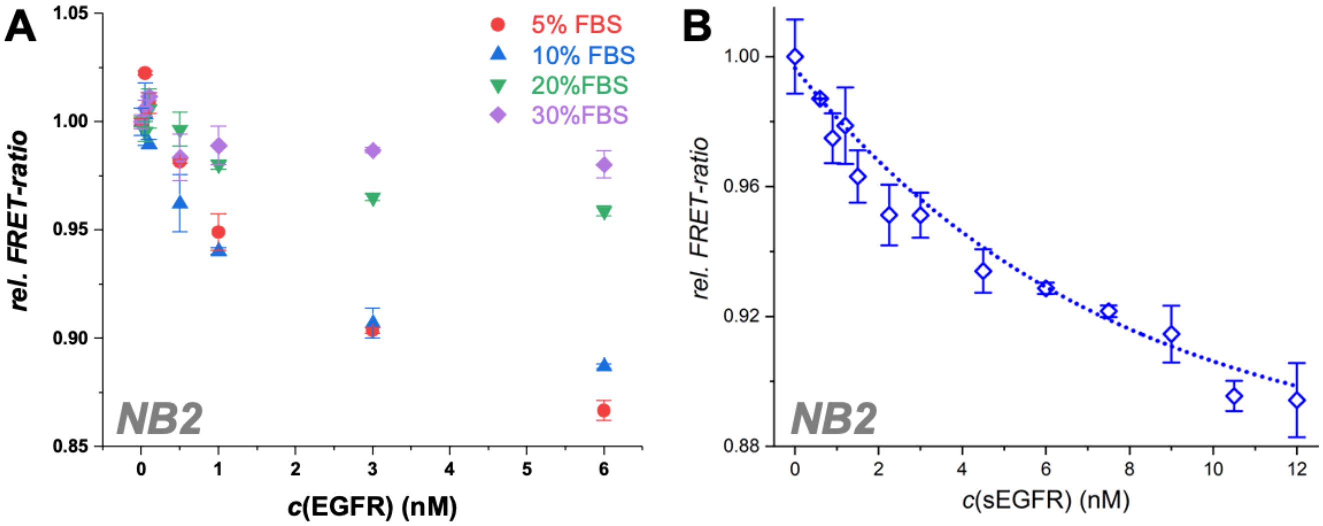

Figure S11. Influence of different NBs. **A:** Relative FRET-ratios of the NB2+QD system against different sEGFR concentrations (0.05, 0.1, 0.5, 1, 3, 6 nM respectively) in 50 µL buffer containing different amount of FBS (5%, 10%, 20%, 30%). **B:** NB displacement FRET immunoassay calibration curves of assay mixtures containing 50 µL of different sEGFR concentrations (in 50 µL buffer containing 10 % FBS), 50 µL of 0.05 nM QD625-CL4, and 50 µL of 3 nM Tb-NH2-H. For all measurements, incubation time was 90 min. Data points represent the measurement of three independent samples. Error bars represent standard deviations.

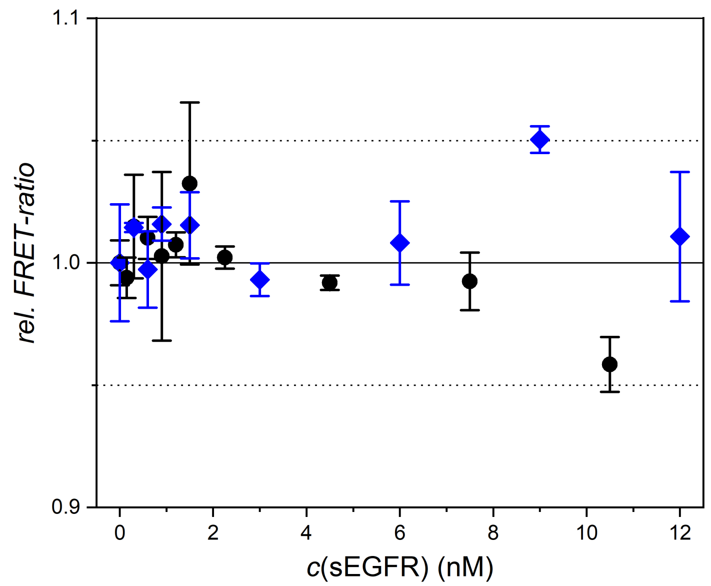

Figure S12. sEGFR-specificity evaluation of NB1 (black) and NB2 (blue) based displacement FRET assay against HER2. FRET-ratio signal changes for HER2 are negligible.

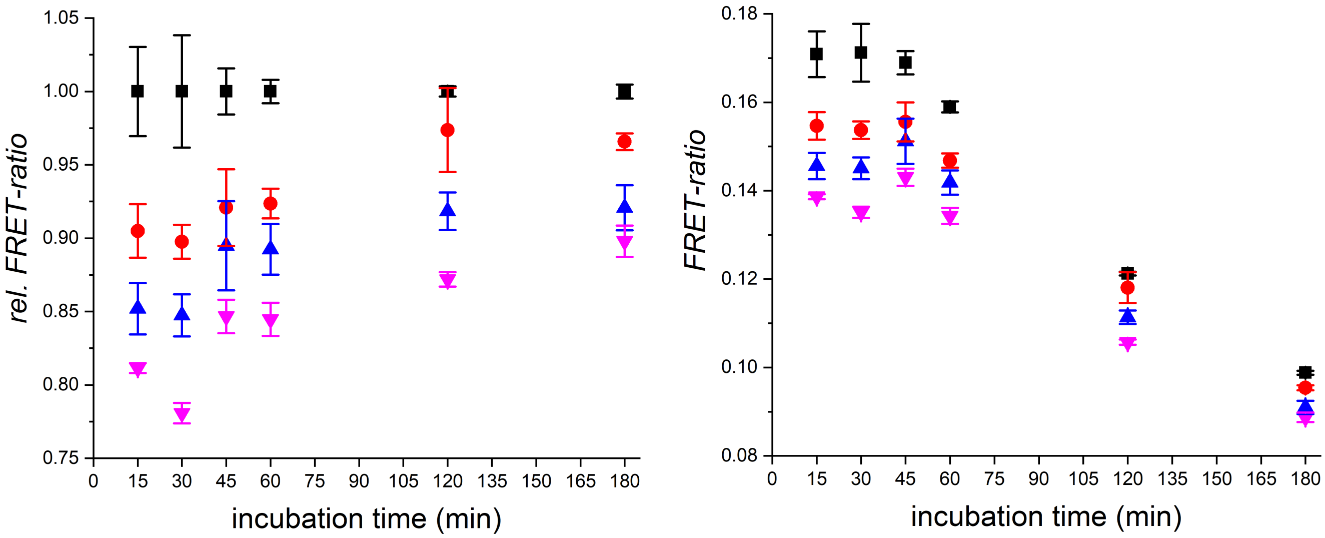

Figure S13. Incubation time dependence of the NB displacement FRET assay. Although the relative (left) and absolute (right) FRET ratios slightly change over time (which is not problematic because of the ratiometric analysis), incubation for 15 or 30 min is sufficient for quantifying EGFR concentration differences.

References

(1) Hendrickson, W. A.; Pähler, A.; Smith, J. L.; Satow, Y.; Merritt, E. A.; Phizackerley, R. P. Crystal Structure of Core Streptavidin Determined from Multiwavelength Anomalous Diffraction of Synchrotron Radiation. *PNAS* **1989**, *86* (7), 2190–2194. https://doi.org/10.1073/pnas.86.7.2190.

(2) Guo, J.; Qiu, X.; Mingoes, C.; Deschamps, J. R.; Susumu, K.; Medintz, I. L.; Hildebrandt, N. Conformational Details of Quantum Dot-DNA Resolved by Forster Resonance Energy Transfer Lifetime Nanoruler. *ACS Nano* **2019**, *13* (1), 505–514. https://doi.org/10.1021/acsnano.8b07137.

(3) Hofman, E. G.; Ruonala, M. O.; Bader, A. N.; van den Heuvel, D.; Voortman, J.; Roovers, R. C.; Verkleij, A. J.; Gerritsen, H. C.; van Bergen en Henegouwen, P. M. P. EGF Induces Coalescence of Different Lipid Rafts. *Journal of Cell Science* **2008**, *121* (15), 2519–2528. https://doi.org/10.1242/jcs.028753.

(4) Zeronian, M. R.; Doulkeridou, S.; van Bergen en Henegouwen, P. M. P.; Janssen, B. J. C. Structural Insights into the Non-Inhibitory Mechanism of the Anti-EGFR EgB4 Nanobody. *under revision* **2022**.

(5) Schmitz, K. R.; Bagchi, A.; Roovers, R. C.; van Bergen en Henegouwen, P. M. P.; Ferguson, K. M. Structural Evaluation of EGFR Inhibition Mechanisms for Nanobodies/VHH Domains. *Structure* **2013**, *21* (7), 1214–1224. https://doi.org/10.1016/j.str.2013.05.008.

(6) Susumu, K.; Oh, E.; Delehanty, J. B.; Blanco-Canosa, J. B.; Johnson, B. J.; Jain, V.; Hervey, W. J.; Algar, W. R.; Boeneman, K.; Dawson, P. E.; Medintz, I. L. Multifunctional Compact Zwitterionic Ligands for Preparing Robust Biocompatible Semiconductor Quantum Dots and Gold Nanoparticles. *J. Am. Chem. Soc.* **2011**, *133* (24), 9480–9496. https://doi.org/10.1021/ja201919s.
